# Treatment of Mitochondrial Disturbances due to Early Life Adversity in Mice Results in Restoration of Complex I Activity and Normal Reward Behavior

**DOI:** 10.1523/ENEURO.0172-25.2025

**Published:** 2025-09-24

**Authors:** Kathie L. Eagleson, Pat Levitt

**Affiliations:** ^1^Division of Neurology, Developmental Neuroscience and Neurogenetics Program, Children’s Hospital Los Angeles, The Saban Research Institute, Los Angeles, California 90027; ^2^Department of Pediatrics, Keck School of Medicine of University of Southern California, Los Angeles, California 90027

**Keywords:** anhedonia, early life adversity, mitochondria, sex differences

## Abstract

The environment experienced by children, such as exposure to chronic early life adversity (ELA), increases lifespan brain disorder risk. The mechanisms that link ELA exposure to functional brain disruptions are not well understood. A limited-bedding and nesting paradigm, in which ELA is induced in mouse pups over the first postnatal week through disruption of maternal care, is characterized by limited resources, environment unpredictability, and disruption of reward and cognitive behaviors. Studies using this model demonstrated sex-selective alterations in hippocampal mitochondrial-associated proteins in response to ELA compared with care as usual (CAU). Further, oxidative phosphorylation (OXPHOS) capacity and complex I activity are increased in ELA juveniles, yet decreased in adults, with the impact of ELA moderated by sex in adults. Given that altered mitochondrial function is a key mediator in metabolic adaptations, the goal of the present study was to evaluate the possibility of reversing mitochondrial dysfunction and the anhedonia that accompanies ELA by addressing oxidative stress. Treatment with the antioxidant MitoQ began at weaning and extended to 3 months. Measures of complex I activity demonstrated full recovery in adults. Female-specific deficits in the sucrose preference task, which is a measure of rewarding behavior in rodents, also exhibited recovery, with preference for sucrose comparable with that of CAU mice. These data indicate that mitochondrial health is one component of responses to early life adversity that has lifespan implications, but with the capacity to recover normal functioning in adults.

## Significance Statement

Chronic early life stress in humans and animals leads to enduring functional changes to brain circuitry and behavior. An emerging concept involves the adaptation of mitochondria to address new energy demands as a primary cellular response to chronic stress. Long-term disruptions lead to oxidative stress, anhedonia, and cognitive deficits. To determine if direct treatment of oxidative stress can mitigate adult dysfunction, an 8 week regimen of an antioxidant was administered to postweaning mice that were raised early postnatally in an environment that disrupts predictable maternal care. Complex I mitochondrial energetics and sucrose preference were restored to levels comparable with mice raised in care as usual conditions, demonstrating a link between mitochondrial health and functional outcomes.

## Introduction

The early environment experienced by infants and toddlers, such as exposure to adverse childhood experiences [ACEs, a form of early life adversity (ELA)], has a pronounced impact on brain disorder and disease risk (Newnham and Ross, 2009; Gluckman et al., 2010). Some cognitive, social-emotional, reward, and physical health disturbances due to ELA are first evident in childhood (Duke and Borowsky, 2018; Cprek et al., 2020; Gilgoff et al., 2020), but others emerge during adolescence and across the lifespan (Felitti et al., 1998; Anda et al., 1999, 2006; Dube et al., 2003, 2009; Edwards et al., 2003; Danese et al., 2009). Thus, ELA contributes significantly to higher disease burden, health disparities, and healthcare costs of over $600 billion annually (Greenberg et al., 2015; Waters and Graf, 2018). The clinically documented disturbances in cognition and mental health (Deschenes et al., 2018; Duke and Borowsky, 2018; Horn et al., 2019; Cprek et al., 2020; Gateau et al., 2023) are hypothesized to be due in part to metabolic maladaptations (Picard et al., 2017; Picard and McEwen, 2018; Ruigrok et al., 2021b; Zitkovsky et al., 2021), but the mechanisms that link ELA exposure to the appearance of clinical symptoms at different times across the lifespan are not well understood. Based on converging evidence from preclinical stress models and ELA-exposed clinical populations, it has been postulated that altered mitochondrial function and the accompanying increases in oxidative stress are key mediators in metabolic maladaptations and the emergence of ELA-induced altered brain physiology (Hoffmann and Spengler, 2018; Picard and McEwen, 2018; Daniels et al., 2020; Eagleson et al., 2020; Zitkovsky et al., 2021; Chaudhari et al., 2022).

To address the knowledge gap in mechanisms that underlie the impact of ELA on long-term brain and body health risks, a limited-bedding and nesting paradigm (LBN) in rodents, in which ELA is induced in mouse pups over the first postnatal week through disruption of maternal care that produces unpredictable interactions with pups (Walker et al., 2017; Birnie et al., 2022), is highly relevant to human ACEs (e.g., neglect, limited resources, unpredictability). A comparative proteomic study using this model demonstrated sex-selective alterations in hippocampal mitochondrial-associated proteins in response to ELA, with the number of altered proteins increasing over time (Eagleson et al., 2020). Further, the study reported that oxidative phosphorylation (OXPHOS) capacity and complex I activity were increased in ELA juveniles, yet decreased in adults, with the impact of ELA moderated by sex in adults only. The mitochondrial electron transport chain, principally complex I, is a major generator of cellular reactive oxygen species (ROS), with increased activity associated with increased ROS production (Cadenas and Davies, 2000; Liu et al., 2002; Turrens, 2003; Brand, 2010). Observational studies in humans and experiments in animal models thus strongly suggest that altered mitochondrial function is a key mediator in metabolic adaptations in development and aging processes that may underlie eventual dysfunctions and more rapid senescence (Bobba-Alves et al., 2023; Monzel et al., 2024; Shaulson et al., 2024).

To our knowledge, studies have not been performed to determine the possibility of reversing mitochondrial dysfunction and the anhedonia that accompanies ELA by addressing oxidative stress. Treatment with the antioxidant mitoquinone mesylate (MitoQ) has been used to reverse oxidative stress in models of adult peripheral organ dysfunction (Lim et al., 2011; Fouret et al., 2015; Gioscia-Ryan et al., 2018; Chen et al., 2020). Further, because MitoQ crosses the blood–brain barrier (Murphy and Smith, 2007), it also has been used to mitigate brain disturbances (Adlam et al., 2005; McManus et al., 2011; Mao et al., 2013; Santini et al., 2015; Stucki et al., 2016; Maiti et al., 2018; Young and Franklin, 2019; Jeong et al., 2021; Haidar et al., 2022). Thus, we posed a basic question: can antioxidant treatment at weaning through early adulthood, readily administered in drinking water, reverse mitochondrial dysfunction and a well-described behavioral disturbance—anhedonia? Adult hippocampal measures of complex I activity were performed in control- and MitoQ-treated mice raised in a LBN environment early postnatally and compared with care-as-usual (CAU) mice. Offspring performance on the sucrose preference task, which is a measure of rewarding behavior in rodents, was evaluated following the treatment regimen. The data indicate that mitochondrial health is one component of responses to ELA that has lifespan implications, but with the capacity to recover normal functioning in adults.

## Materials and Methods

**Figure 1.**
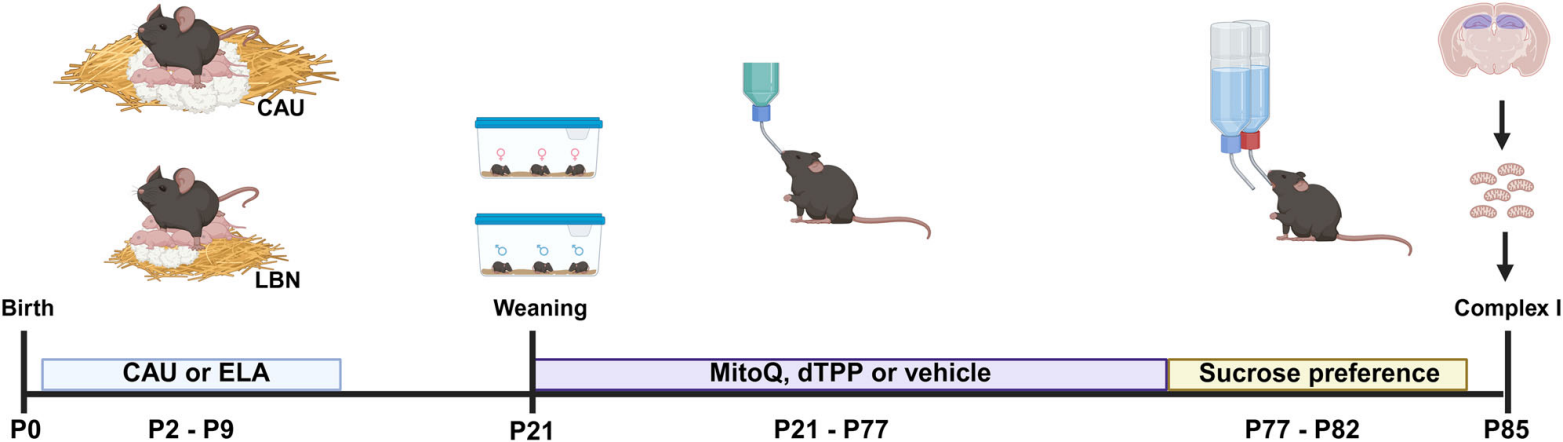
Study design. Litters were assigned to their experimental group at postnatal day (P) 0, the day of birth. On P2, litters were culled to sex-balanced six pups and moved to either a new care-as-usual (CAU) cage or to a limited bedding and nesting (LBN) cage. The LBN paradigm induces early life adversity (ELA) in the pups. All litters were returned to a fresh CAU cage at P9, underwent a cage change at P16, and were weaned and housed with same sex littermates at P21. Starting at weaning, ELA mice were maintained on either MitoQ, dTPP or vehicle in drinking water for 8 weeks. CAU mice received vehicle only. At the end of the treatment period, mice first were tested for sucrose preference, then, at least 3 d later, hippocampal complex I activity was determined. Created in BioRender. https://BioRender.com/a02d73k.

The study design is outlined in Figure 1. Details of experimental cohorts are provided in Table 1.

**Table 1.** Experimental cohorts used in study

Cohort	CAU + vehicle	ELA + vehicle	ELA + dTPP	ELA + MitoQ
	DOB (M:F)	DOB (M:F)	DOB (M:F)	DOB (M:F)
1	3/15/21 (3:3)	3/15/21 (3:3)	3/15/21 (3:3)	3/16/21 (3:3)
2	3/20/21 (3:3)	3/22/21 (3:3)	3/22/21 (2:4)	3/27/21 (3:3)
3	5/28/21 (3:3)	5/28/21 (3:3)	5/28/21 (3:3)	5/29/21 (3:3)
4	6/1/21 (2:4)	6/6/21 (4:2)	6/7/21 (3:3)	6/7/21 (3:3)
5	6/8/21 (3:3)	6/11/21 (3:3)	6/12/21 (3:3)	6/13/21 (3:3)
6	6/25/23 (3:3)	6/25/23 (3:3)	6/26/23 (3:3)	7/1/23 (2:4)
7	7/6/23 (3:3)	7/10/23 (3:3)	7/12/23 (3:3)	7/13/23 (3:3)
8	8/25/23 (2:4)	8/26/24 (3:3)	8/30/24 (3:3)	8/30/24 (2:4)
9	9/19/23 (3:3)	9/14/23 (3:3)	9/15/23 (3:3)	9/19/23 (3:3)
10	11/1/23 (3:3)	11/1/23 (3:3)	11/2/23 (3:3)	11/8/23 (3:3)
11	11/9/23 (3:3)	11/11/23 (3:3)	11/12/23 (3:3)	11/16/23 (3:3)
12	1/10/24 (3:3)	1/9/24 (3:3)	1/10/24 (3:3)	1/12/24 (3:3)
13	2/2/24 (3:3)	2/3/24 (3:3)	2/4/24 (3:3)	2/9/24 (3:3)
14	3/6/24 (3:3)	3/6/24 (3:3)	3/7/24 (3:3)	3/8/24 (3:3)
15	3/8/24 (3:3)	3/9/24 (3:3)	3/9/24 (3:3)	3/9/24 (3:3)

CAU, care-as-usual; ELA, early life adversity; DOB, date of birth; M, male; F, female.

### Mice and animal husbandry

All mouse procedures were approved by the Institutional Animal Care and Use Committee at the Children's Hospital Los Angeles and conformed to NIH guidelines. Mice were housed in Super Mouse 750 ventilated cages (#75050PC-GAW, Lab Products) that included a LifeSpan Rodent Enrichment module (#75009PC, Lab Products) in a temperature- and humidity-controlled vivarium under at 13/11 h light/dark cycle. Food (PicoLab Rodent Diet 20, #5053) and water were provided *ad libitum*. Cages were changed weekly and provided with 100 g bedding (P.J. Murphy Forest Products Sani-Chips, #91100, Newco Distributors) and one standard 2″ nestlet square (#6002, Newco Distributors).

Male and female C57BL/6J mice (RRID: IMSR_JAX:000664) were shipped from the Jackson Laboratory at 8–10 weeks of age. Mice were acclimated to the colony for 5 d, and then breeding was initiated by housing four females with one male. After 14 d, breeding cages were monitored daily, and pregnant dams removed and housed singly until pups were born. The breeding cycle was then repeated to generate second litters, with all experiments performed on the second litter of each dam. Sixty litters generated between March 2021 and March 2024 were used in the current study, with 15 litters representing one of four experimental groups (see below).

### ELA paradigm

A modified limited-bedding and nesting (LBN) paradigm, developed in the Baram laboratory (Rice et al., 2008), was used to induce ELA in mice. Each cohort comprised four litters, each representing one experimental group that had been assigned sequentially based on day of birth, designated postnatal day (P) 0 (Table 1). On P2, litters were culled to six pups (sex ratio 3:3 or 4:2) and exposed to CAU or ELA rearing. CAU litters (*n* = 15) were maintained on standard nesting and bedding material. For ELA litters (*n* = 45), the floor of the cage was covered with 50 g of bedding, and a stainless steel raised wire floor with 10 × 10 mm square openings and 1 mm wire diameter (#RWF7JMV) was inserted above the cage floor. One-half (∼14 g) of a standard nestlet square was provided and the LifeSpan Rodent Enrichment module removed. Both cage setups have identical access to food and water. ELA and CAU litters were placed in new CAU cages on P9, cage changes carried out on P16, and litters weaned and housed with same-sex littermates on P21.

### MitoQ intervention

Mice were maintained on a MitoQ regimen starting at weaning, extending through puberty and adolescence into young adulthood. During the synthesis of MitoQ, the ubiquinol antioxidant is attached to a lipophilic triphenylphosphonium (TPP) cation to facilitate selective targeting to mitochondria. Decyltriphenylphosphonium (dTPP) is identical structurally to MitoQ but lacks the ubiquinone moiety. We used dTPP alone to control for potential effects of the TPP cation, which has been reported in other studies (Gottwald et al., 2018; Armstrong et al., 2019). ELA mice were treated for 8–9 weeks with either vehicle (dimethylsulfoxide, DMSO, #D8418, Sigma-Aldrich), 100 μM MitoQ (#317102, MedKoo Biosciences), or 100 μM dTPP (#620110, MedKoo Biosciences) in drinking water. CAU mice were administered vehicle only, representing the control group for comparison. This design has been used in multiple preclinical studies examining the effect of MitoQ in mitigating negative outcomes associated with experimental manipulations and disease models, including high-fat diet, traumatic brain injury, spinal cord injury, acute pancreatitis, melanoma, and Alzheimer's (Huang et al., 2015, 2022; Bond et al., 2019; Young and Franklin, 2019; Le Gal et al., 2021; Haidar et al., 2022). The drinking water was refreshed weekly. Thus, the four experimental groups are (1) CAU plus vehicle (CAU + Veh), (2) ELA plus vehicle (ELA + Veh), (3) ELA plus dTPP (ELA + dTPP), and (4) ELA plus MitoQ (ELA + MitoQ).

### Sucrose preference test

At the end of the treatment period, mice underwent a sucrose preference test according to a published protocol (Goodwill et al., 2019). To avoid isolation stress, mice were housed in littermate pairs in a N10 high-temp mouse cage with an N10SS mouse lid (Ancare) for the duration of the protocol. Mice were habituated for 48 h to the new cage and the two-bottle paradigm. Over this period, both bottles (each 100 ml) contained drinking water. After habituation, drinking water in one bottle was replaced with 1% sucrose in drinking water and mice were given a free choice between the two bottles over a 72 h test period. To control for potential side-preference effects, the position of the bottles in the cage were switched every 24 h. Bottles were weighed before and after the test period to calculate water and sucrose intake. Sucrose preference was calculated as the percentage of sucrose solution consumed relative to the total amount of liquid drunk during the 72 h test (sucrose solution intake / total intake × 100), generating a single value for each cage.

### Complex I activity

At least 3 d after completing the sucrose preference task, mice were anesthetized with 4% isoflurane (#1169567772, Covetrus) and decapitated and the hippocampus dissected. Hippocampal tissue (∼100 mg) from three male or three female pups within a single litter was pooled to generate a single biological sample. This amount of starting tissue, rapidly dissected to avoid impacting mitochondrial health during isolation (Liao et al., 2020), is necessary to obtain highest quality data using the enzyme-linked immunosorbent assay (ELISA) mitochondrial complex I assay across a range of protein concentrations and controls. Mitochondria were isolated using the MITOISO1 mitochondrial isolation kit (#MITOISO1, Sigma-Aldrich), following the manufacturer's protocol. Mitochondrial protein content was determined using the DC Protein Assay Kit II (#5000112, Bio-Rad). Mitochondrial complex I activity was measured using an immuno-capture ELISA complex I enzyme activity assay kit, according to the manufacturer's protocol (#ab109721, Abcam). To ensure that all assays were in the linear range, three dilutions of mitochondrial proteins (20–80 μg) from each sample were plated per well. Measurements were done in duplicates that were averaged. Activity was expressed as change in absorbance/minute/mg protein. Complex I data are not presented for cohorts 1 and 11 (low levels of protein after mitochondrial isolation) and cohort 6 (Complex I kit unavailable due to quality control issues).

### Statistical analyses

Data are reported as mean [95% confidence interval (CI)]. Individual measures for each assay are reported in the graphs and Extended Data Figures. For each assay and sex, an individual litter represents a single sample. For analyses of offspring body weight, weights and percentage weight gain of male or female pups were averaged across a litter to generate a single value for each sex in each litter at each age. The study was designed to detect medium to large effects in complex I activity and sucrose preference at *α* = 0.05 and 1−*β* = 0.80. Effects sizes were determined by calculating eta-squared (*η*^2^), with small, medium, and large effects classified as 0.010, 0.060, and >0.140, respectively. Power analyses were conducted to determine sample sizes, using means and standard deviations observed in previous studies using G*Power (Heinrich-Heine-Universitat Dusseldorf).

For preweanling weight measures, comparisons between groups were performed using an unbalanced two-way analysis of variance (ANOVA), with sex and ELA as between-subjects factors. For adult weight and complex I activity measures, comparisons between groups were performed using a balanced two-way ANOVA, with sex and treatment as between-subjects factors. Significant differences with large to medium effect sizes were followed up with Tukey’s honestly significant difference (HSD) test to identify the source of possible interactions. If no significant sex or sex × ELA/sex × treatment interaction was observed, data were collapsed across sex for post hoc analyses. If a significant sex or sex × ELA/sex × treatment interaction was observed, post hoc analyses considered males and females independently. For sucrose preference measures, the impact of dTPP and MitoQ treatment was determined only in females using a one-way ANOVA followed by a Tukey HSD test. For all measures, a Hedge's *g*, appropriate for sample sizes <30, was calculated as an estimate of effect size for vehicle-treated ELA compared with vehicle-treated CAU mice, and for dTPP- and MitoQ-treated compared with vehicle-treated ELA and vehicle-treated CAU mice, with small, medium, and large effects classified as 0.2, 0.5, and >0.8, respectively. For all tests, *p* values are reported to the third decimal place; a priori *α* = 0.050. Final sample sizes, effect sizes, *F* statistics, and *p* values are reported in Tables 2 and 3.

**Table 2.** Statistical test details for body weight measures

Figure	Measure, test	Factors	Test statistic	*p* value	Effect size
	P2 weight (CAU: *n* = 30, ELA: *n* = 90; Females: *n* = 60, Males: *n* = 60)				
	Unbalanced two-way ANOVA	Sex	*F*_(1,116)_ = 2.812	0.096	*η*^2^ = 0.024
		ELA	*F*_(1,116)_ = 0.097	0.756	*η*^2^ = 0.001
		Sex × ELA	*F*_(1,116)_ = 0.246	0.621	*η*^2^ = 0.002
2*A*	% weight gain P2–P9 (CAU: *n* = 30, ELA: *n* = 90; Females: *n* = 60, Males: *n* = 60)				
	Unbalanced two-way ANOVA	Sex	*F*_(1,116)_ = 0.303	0.583	*η*^2^ = 0.003
		ELA	*F*_(1,116)_ = 53.178	<0.001	*η*^2^ = 0.310
		Sex × ELA	*F*_(1,116)_ = 0.104	0.748	*η*^2^ = 0.001
2*B*	P9 weight (CAU: *n* = 30, ELA: *n* = 90; Females: *n* = 60, Males: *n* = 60)				
	Unbalanced two-way ANOVA	Sex	*F*_(1,116)_ = 3.169	0.078	*η*^2^ = 0.027
		ELA	*F*_(1,116)_ = 110.365	<0.001	*η*^2^ = 0.490
		Sex × ELA	*F*_(1,116)_ = 0.164	0.686	*η*^2^ = 0.001
2*C*	% weight gain P9–P21 (CAU: *n* = 30, ELA: *n* = 90; Females: *n* = 60, Males: *n* = 60)				
	Unbalanced two-way ANOVA	Sex	*F*_(1,116)_ = 51.787	<0.001	*η*^2^ = 0.310
		ELA	*F*_(1,116)_ = 34.700	<0.001	*η*^2^ = 0.230
		Sex × ELA	*F*_(1,116)_ = 16.702	<0.001	*η*^2^ = 0.130
	Post hoc (Tukey’s HSD)	Female CAU, female ELA		0.328	*g* = 0.459
		Male CAU, male ELA		0.004	*g* = 1.197
2*D*	P21 weight (CAU: *n* = 30, ELA: *n* = 90; Females: *n* = 60, Males: *n* = 60)				
	Unbalanced two-way ANOVA	Sex	*F*_(1,116)_ = 8.533	0.004	*η*^2^ = 0.069
		ELA	*F*_(1,116)_ = 25.026	<0.001	*η*^2^ = 0.180
		Sex × ELA	*F*_(1,116)_ = 0.546	0.462	*η*^2^ = 0.005
	Post hoc (Tukey’s HSD)	Female CAU, female ELA		<0.001	*g* = 1.174
		Male CAU, male ELA		0.016	*g* = 0.937
3*A*	P21 ELA weight (*n* = 15/group)				
	Two-way ANOVA	Sex	*F*_(1,84)_ = 8.145	0.005	*η*^2^ = 0.088
		Treatment	*F*_(2,84)_ = 0.965	0.385	*η*^2^ = 0.022
		Sex × Treatment	*F*_(2,84)_ = 0.215	0.807	*η*^2^ = 0.005
	Post hoc (one-way ANOVA)	Female	*F*_(2,42)_ = 0.959	0.391	*η*^2^ = 0.044
		Male	*F*_(2,42)_ = 0.196	0.823	*η*^2^ = 0.097
3*B*	Adult weight (*n* = 15/group)				
	Two-way ANOVA	Sex	*F*_(1,112)_ = 765.5487	<0.001	*η*^2^ = 0.870
		Treatment	*F*_(3,112)_ = 0.3661	0.778	*η*^2^ = 0.009
		Sex × Treatment	*F*_(3,112)_ = 0.3379	0.798	*η*^2^ = 0.009
	Post hoc (one-way ANOVA)	Female	*F*_(2,42)_ = 0.008	0.992	*η*^2^ = 0.001
		Male	*F*_(2,42)_ = 0.237	0.790	*η*^2^ = 0.110

**Table 3.** Statistical test details for complex I and sucrose preference measures

Figure	Measure, test	Factors	*F* statistic	*p* value	Effect size
4	Complex I activity (*n* = 12/group)				
	Two-way ANOVA	Sex	*F*_(1,88)_ = 4.973	0.028	*η*^2^ = 0.053
		Treatment	*F*_(3,88)_ = 17.920	<0.001	*η*^2^ = 0.380
		Sex × treatment	*F*_(3,88)_ = 1.249	0.297	*η*^2^ = 0.047
	Post hoc—female (Tukey’s HSD)	CAU (veh), ELA (veh)		<0.001	*g* = 2.777
		ELA (veh), ELA (dTPP)		0.517	*g* = 0.551
		ELA (veh), ELA (MitoQ)		<0.001	*g* = 2.336
		CAU (veh), ELA (dTPP)		<0.001	*g* = 1.947
		CAU (veh), ELA (MitoQ)		0.799	*g* = 0.380
	Post hoc—male (Tukey’s HSD)	CAU (veh), ELA (veh)		0.017	*g* = 1.221
		ELA (veh), ELA (dTPP)		0.999	*g* = 0.059
		ELA (veh), ELA (MitoQ)		0.302	*g* = 0.764
		CAU (veh), ELA (dTPP)		0.024	*g* = 1.143
		CAU (veh), ELA (MitoQ)		0.551	*g* = 0.499
5	Female sucrose preference test (*n* = 15/group)				
	One-way ANOVA		*F*_(3,56)_ = 21.728	<0.001	*η*^2^ = 0.540
	Post hoc (Tukey’s HSD)	CAU (veh), ELA (veh)		<0.001	*g* = 3.062
		ELA (veh), ELA (dTPP)		<0.001	*g* = 1.349
		ELA (veh), ELA (MitoQ)		<0.001	*g* = 3.187
		CAU (veh), ELA (dTPP)		0.045	*g* = 0.841
		CAU (veh), ELA (MitoQ)		0.988	*g* = 0.161

## Results

**Figure 2.**
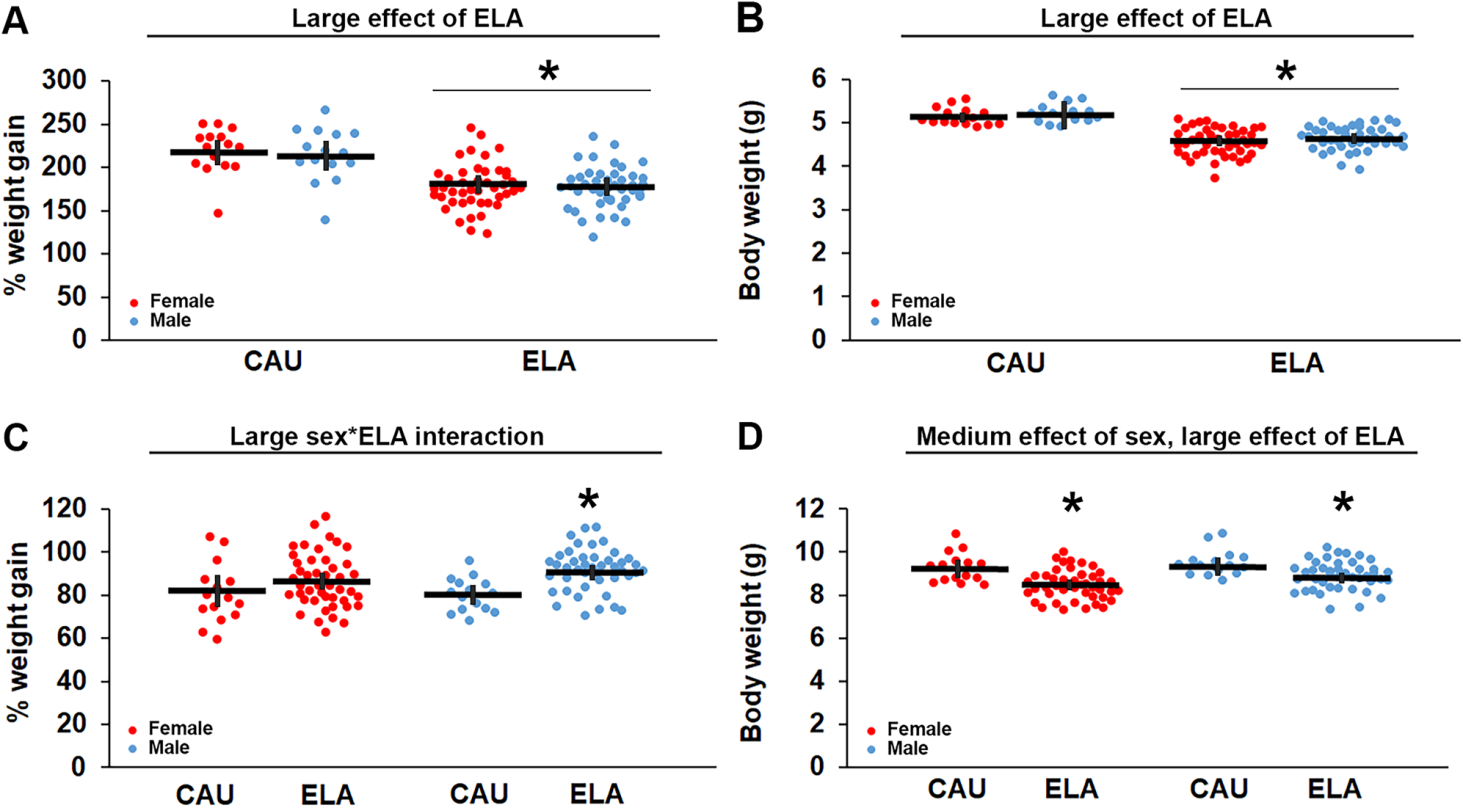
Early life adversity (ELA) impacts preweanling body weight gain. ***A***, Percent body weight gain over the P2–9 ELA period is significantly reduced in female and male ELA mice compared with care-as-usual (CAU) female and male mice. ***B***, ELA pups weigh significantly less than CAU pups at P9. ***C***, Percent body weight gain is significantly increased in male ELA pups, but not female ELA pups, after the ELA period ends. ***D***, ELA pups weigh less than CAU pups at weaning. *Significantly different to CAU. Statistical details and samples sizes are provided in Table 2. The raw data contributing to this figure are included in Extended Data Figure 2-1. Each symbol represents an independent sample, with the bars representing the group mean ± 95% confidence interval. Red circles, females. Blue circles, males.

**Figure 3.**
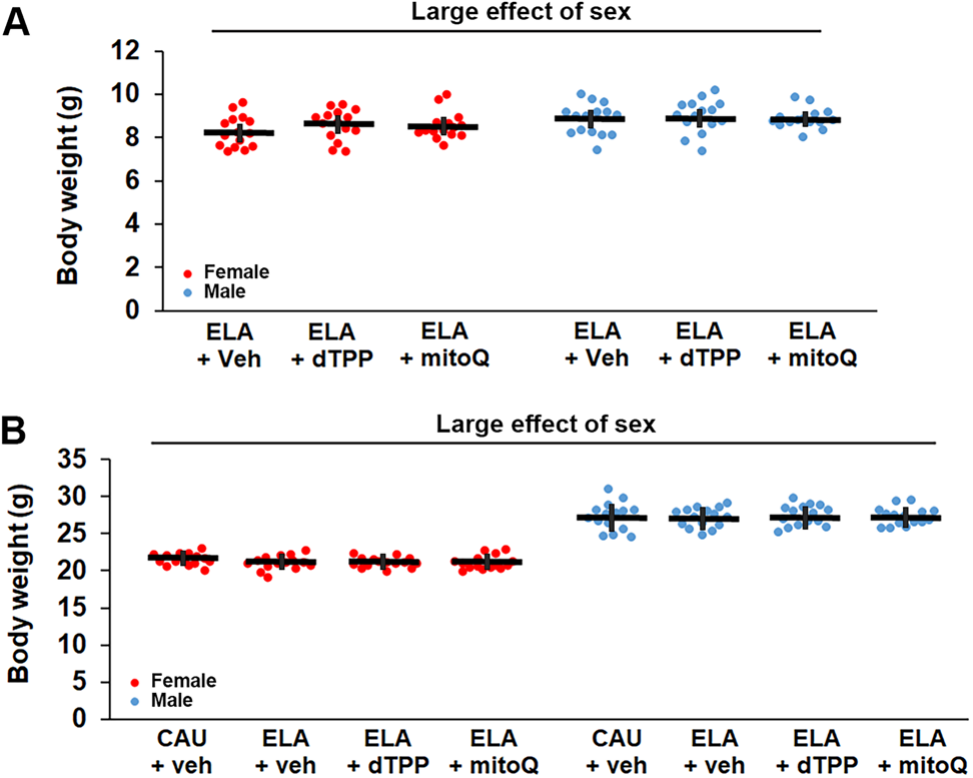
There is no difference in body weight across mice assigned to each early life adversity (ELA) treatment group. ***A***, Body weights across mice assigned to the vehicle (veh), dTPP, and MitoQ treatment groups are not significantly different for males or females at P21, the start of the treatment paradigm. ***B***, Treatment with dTPP and MitoQ has no effect on adult body weight compared with vehicle-treated ELA mice at the start of the behavior study. Statistical details and samples sizes are provided in Table 2. The raw data contributing to this figure are included in Extended Data Figure 3-1. Each symbol represents an independent sample, with the bars representing the group mean ± 95% confidence interval. Red circles, females. Blue circles, males.

A consistent outcome of the LBN paradigm is reduced pup weight gain over the ELA period. We used pup weight measures to validate the impact of the ELA manipulation (Rice et al., 2008) and to confirm there were no differences in the weights of pups assigned to the ELA treatment groups prior to initiation of treatment at P21 or outcome measures in adults (Extended Data Fig. 2-1). There was no significant difference in pup weight between litters assigned to CAU or ELA at P2, the start of the ELA period (Table 2; CAU: 1.66 g, 95% CI [1.57, 1.75], ELA: 1.67 g, 95% CI [1.62, 1.72]). There was a significant large effect of ELA (*η*^2^ = 0.310), but no significant effect of sex or sex × ELA interaction, on weight gain over the P2–P9 ELA period (Fig. 2*A*, Table 2). Specifically, there was a significant reduction in percent body weight gain in ELA (177.54%, 95% CI [171.33, 181.69]) compared with CAU (216.32%, 95% CI [205.65, 226.99]) mice. Thus, ELA pups weighed ∼12% less than CAU pups at P9 (Fig. 2*B*; CAU: 5.17 g, 95% CI [5.09, 5.25], ELA: 4.53 g, 95% CI [4.35, 4.71]), with no significant effect of sex or sex × ELA interaction (Table 2). There was a significant large effect of ELA (*η*^2^ = 0.230) on weight gain between the end of the ELA period and weaning (Fig. 2*C*, Table 2). Unexpectedly, there was also a large effect of sex (*η*^2^ = 0.310) and sex × ELA interaction (*η*^2^ = 0.130) on this measure; thus, we performed post hoc analyses on males and females independently (Table 2). There was a significant increase in percent body weight gain in male ELA (91.90%, 95% CI [88.60, 95.20]) compared with male CAU (79.83%, 95% CI [75.49, 84.17]) mice. In contrast, there was no significant difference in percent body weight gain in female ELA (86.45%, 95% CI [82.67, 90.23]) compared with female CAU (80.51%, 95% CI [72.76, 88.26]) mice. Thus, there was a medium effect of sex (*η*^2^ = 0.069) and a large effect of ELA (*η*^2^ = 0.180) on pup weight at weaning (Fig. 2*D*, Table 2), with ELA having a larger effect in females (Hedge's *g* = 1.174; CAU: 9.27 g, 95% CI [8.89, 9.65], ELA: 8.46, 95% CI [8.25, 8.67]) than males (Hedge's *g* = 0.37; CAU: 9.47 g, 95% CI [9.13, 9.81], ELA: 8.87 g, 95% CI [8.67, 9.07]). Importantly, there was no significant difference in pup weight across ELA litters assigned to different treatment groups at P21, when treatment was initiated, in females (ELA + vehicle: 8.27 g, 95% CI [7.87, 8.67], ELA + dTPP: 8.59 g, 95% CI [8.20, 8.98], ELA + MitoQ: 8.53 g, 95% CI [8.18, 8.88]) or males (ELA + vehicle: 8.80 g, 95% CI [8.40, 9.20], ELA + dTPP: 8.95 g, 95% CI [8.53, 9.37], ELA + MitoQ: 8.85 g, 95% CI [8.60, 9.10]; Fig. 3*A*, Extended Data Fig. 3-1, Table 2). Similarly, at the start of the behavior study in adults, while there is the expected significant large effect of sex on body weight (*η*^2^ = 0.067; females: 21.17 g, 95% CI [20.94, 21.39]; males: 27.20 g, 95% CI [26.83, 27.57]), there was no effect of treatment on ELA body weight in females (ELA + vehicle: 21.03 g, 95% CI [20.51, 21.55], ELA + dTPP: 21.08 g, 95% CI [20.70, 21.46], ELA + MitoQ: 21.06 g, 95% CI [20.55, 21.57]) or males (ELA + vehicle: 27.05 g, 95% CI [26.32, 27.78], ELA + dTPP: 27.38 g, 95% CI [26.62, 28.14], ELA + MitoQ: 27.19 g, 95% CI [26.53, 27.85]; Fig. 3*B*, Extended Data Fig. 3-1, Table 2).

10.1523/ENEURO.0172-25.2025.f2-1 Figure 2-1 Preweanling body weight (g) and weight gain (%). Download Figure 2-1, XLS file.

10.1523/ENEURO.0172-25.2025.f3-1 Figure 3-1 Body weight (g) across ELA treatment groups at initiation of treatment (P21) and start of behavior (adult). Download Figure 3-1, XLS file.

### MitoQ mitigates the impact of ELA on adult outcomes

**Figure 4.**
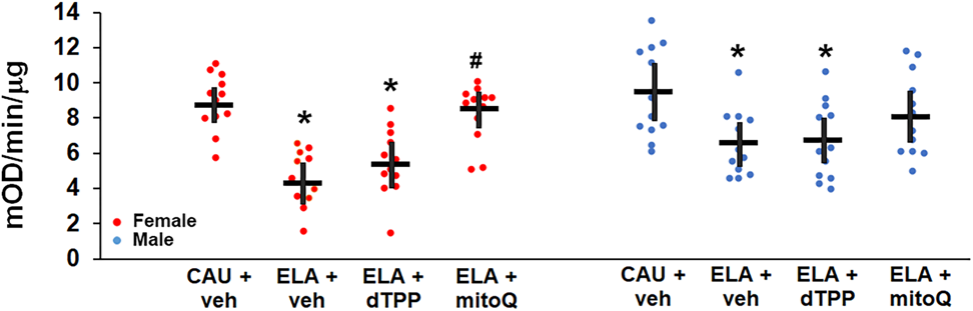
MitoQ treatment mitigates the effect of early life adversity (ELA) on young adult female hippocampal complex I activity. Complex I activity was measured in isolated hippocampal mitochondria. Complex I activity is reduced in ELA mice treated with vehicle (veh), with a larger effect in females compared with males. In females, treatment with MitoQ, but not dTPP, starting at weaning, mitigated the effect of ELA. *Significantly different to care-as-usual (CAU) + veh. ^#^Significantly different to ELA + veh. Statistical details and samples sizes are provided in Table 3. The raw data contributing to this figure are included in Extended Data Figure 4-1. Each symbol represents an independent sample, with the bars representing the group mean ± 95% confidence interval. Red circles, females. Blue circles, males.

There was a significant large effect of treatment (*η*^2^ = 0.380) on hippocampal complex I activity (Fig. 4, Extended Data Fig. 4-1, Table 3). Sex also had a significant effect on hippocampal complex I activity (*η*^2^ = 0.053; Table 3); thus, post hoc analyses were performed on males and females independently. Consistent with previous reports (Eagleson et al., 2020), ELA significantly decreased adult hippocampal complex I activity, with a larger effect size in females (Hedge's *g* = 2.777; CAU + vehicle: 8.91 mOD/min/μg, 95% CI [8.02, 9.80], ELA + vehicle: 4.55 mOD/min/μg, 95% CI [3.70, 5.40]) compared with males (Hedge's *g* = 1.221; CAU + vehicle: 9.42 mOD/min/μg, 95% CI [7.99, 10.85], ELA + vehicle: 6.57 mOD/min/μg, 95% CI [5.34, 7.61]). In females, treatment with MitoQ resulted in a significant improvement in hippocampal complex I activity (ELA + MitoQ: 8.29 mOD/min/μg, 95% CI [7.37, 9.21]), with a large effect size (Hedge's *g* = 1.358, Table 3), such that activity was normalized to CAU + vehicle levels. In contrast, treatment with dTPP had no significant effect (ELA + dTPP: 5.50 mOD/min/μg, 95% CI [4.45, 6.55]). Surprisingly, in males there was no significant impact of dTPP or MitoQ treatment on hippocampal complex I activity (ELA + dTPP: 6.69 mOD/min/μg, 95% CI [5.48, 7.90]; ELA + MitoQ: 8.19 mOD/min/μg, 95% CI [6.89, 9.49]) compared with vehicle-treated ELA mice, likely reflecting higher baseline activity in male ELA mice. We note, however, that there was a medium effect size of MitoQ treatment (Hedge's *g* = 0.765) that was sufficient to normalize male ELA complex I activity to the CAU + vehicle range. Conversely, dTPP treatment had no effect (Hedge's *g* = 0.059), with activity remaining significantly different to CAU + vehicle (Table 3).

10.1523/ENEURO.0172-25.2025.f4-1 Figure 4-1 Complex I data. Download Figure 4-1, XLS file.

**Figure 5.**
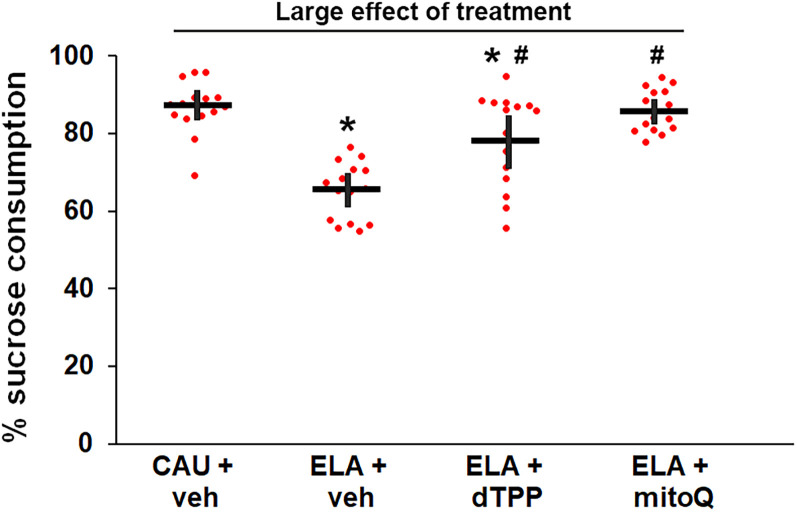
MitoQ treatment mitigates the effect of early life adversity (ELA) on sucrose preference in young adult female mice. Sucrose preference is reduced in ELA mice treated with vehicle (veh). Treatment of ELA mice with dTPP and MitoQ significantly increases sucrose preference compared with vehicle, with MitoQ-treated mice returning to care-as-usual (CAU) levels. Note the high variation in the dTPP group, compared with the MitoQ group, with 6 of the 15 litters exhibiting sucrose preference scores within the range of the vehicle-treated group, with no mitigation. Neither ELA nor dTPP and MitoQ treatment had an impact on sucrose preference in adult male mice (Extended Data Figs. 5-1, 5-2). *Significantly different to CAU + veh. ^#^Significantly different to ELA + veh. Statistical details and samples sizes are provided in Table 3. The raw data contributing to this figure are included in Extended Data Figure 5-3. Each symbol represents an independent sample, with the bars representing the group mean ± 95% confidence interval.

Previous studies have demonstrated a female-selective reduction in sucrose preference in response to LBN in adult mice (Goodwill et al., 2019; Liu et al., 2021; Zheng et al., 2024). We first confirmed there was no significant difference in sucrose preference between adult CAU (86.8%, 95% CI [83.9, 89.7]) and ELA (83.5%, 95% CI [77.6, 89.4]) males (Welch's *t* test: *t*_(20.59)_ = 1.078, *p* = 0.293; *n* = 15/group; Extended Data Figs. 5-1, 5-2). We then determined the impact of MitoQ treatment on sucrose preference in females exposed to ELA. There was a significant large effect of experimental group (*η*^2^ = 0.540) on sucrose preference in adult females (Fig. 5, Extended Data Fig. 5-3, Table 3). ELA significantly reduced sucrose preference with a large effect size (Hedge's *g* = 3.062; CAU + vehicle: 86.9%, 95% CI [83.2, 90.6], ELA + vehicle: 65.3%, 95% CI [61.2, 69.4]). Treatment with MitoQ resulted in a statistically significant improvement in sucrose preference compared with vehicle, with a large effect size (Hedge's *g* = 3.187; Table 3), thus normalizing sucrose preference (85.9%, 95% CI [82.9, 88.9]). Unexpectedly, dTPP treatment also mitigated the impact of ELA on sucrose preference (78.7%, 95% CI [72.0, 85.4]) with a significant large effect size (Hedge's *g* = 1.349). We note that the effect size for dTPP is smaller than that for MitoQ, likely due to the greater variability in the dTPP- compared with MitoQ-treated mice. We then applied the *F* test for equality of variance, revealing a statistically significant, very large difference in variance between ELA + MitoQ (SD = 5.5) and ELA + dTPP (SD = 12.0; *F*_(14,14)_ = 4.76, *p* = 0.006). In fact, 40% of the litters in the ELA + dTPP group were in the same range as the ELA + vehicle group, with no mitigation. This difference is indicative of substantial heterogeneity of the dTPP effect compared with MitoQ for this measure. Finally, we confirmed that MitoQ and dTPP treatment did not impact sucrose preference in male ELA mice (Extended Data Figs. 5-1, 5-2). This suggests that, even in the presence of stress, MitoQ does not cause changes in normal function.

10.1523/ENEURO.0172-25.2025.f5-1 Figure 5-1 Early life adversity (ELA) treatment has no impact on sucrose preference in male mice treated with vehicle (veh), dTPP and MitoQ compared to care-as-usual (CAU; Kruskal Wallis test, H(3) = 4.763, p = 0.190). The raw data contributing to this figure are included in Figure 5-2. Each symbol represents an independent sample, with the bars representing the group mean ± 95% confidence interval. Download Figure 5-1, TIF file.

10.1523/ENEURO.0172-25.2025.f5-2 Figure 5-2 Male sucrose preference test data. Download Figure 5-2, XLS file.

10.1523/ENEURO.0172-25.2025.f5-3 Figure 5-3 Female sucrose preference test data. Download Figure 5-3, XLS file.

## Discussion

The goal of the present study was to address the question of whether mitigating oxidative stress starting at weaning would ameliorate the impact of ELA on young adult physiology and behavior. We evaluated two outcomes: hippocampal complex I activity and sucrose preference, a measure of anhedonia. Mitochondrial dysfunction, including decreased complex I expression and activity, and disrupted reward-based behavior, including depression and anhedonia, are associated with ELA in clinical populations and preclinical animal models (Ridout et al., 2018; Goodwill et al., 2019; Nold et al., 2019; Eagleson et al., 2020; Liu et al., 2021; Ruigrok et al., 2021b; Gyllenhammer et al., 2022; Gorman-Sandler et al., 2023). Our data confirmed reduced complex I activity in the adult hippocampus and the female-specific decrease in sucrose preference previously reported in mice exposed to the LBN model of ELA (Goodwill et al., 2019; Eagleson et al., 2020; Liu et al., 2021; Zheng et al., 2024). We note that, based on several observations, the decrease in sucrose preference in this model has been associated with anhedonia rather than a decrease in metabolism due to a complex I deficiency. First, mitochondrial adaptations to ELA are tissue specific. For example, complex I activity is unaltered in the adult male and female liver and skeletal muscle following LBN (Eagleson et al., 2020; Ruigrok et al., 2021b), indicating there is not a broad decrease in metabolism. Second, in the LBN paradigm, there is no difference in the expression of genes involved in hypothalamic nutrient sensing in adult mice (Ruigrok et al., 2021a) and no overall effect on energy intake in adult female rats (Maniam et al., 2015). The present results further demonstrate that treatment with the mitochondrial antioxidant, MitoQ, prevented these outcomes. Unexpectedly, while dTPP had no effect on complex I activity in adult ELA mice, there was improvement in the sucrose preference test. dTPP is reported to have inconsistent therapeutic effects on its own (see below); the highly variable impact observed here aligns with this. Overall, the mitochondrial and behavioral functional data tested here demonstrate that ELA-induced adult dysfunction can be prevented.

Several approaches have been implemented in the LBN rodent model to improve ELA-induced behavioral dysfunction. Intracerebroventricular infusion of a selective corticotrophin-releasing hormone (CRH) receptor blocker in juveniles or adults (Ivy et al., 2010; Short et al., 2020), systemic administration of a TrkB agonist (7,8-dihydroxyflavone; Sun et al., 2023), or manipulation of the maternal diet during key developmental periods (Naninck et al., 2017; Yam et al., 2019; Geertsema et al., 2024; Reemst et al., 2024) improved hippocampal-dependent cognitive dysfunction. Similarly, anhedonia was reversed following viral injection of *Crh*-shRNA into central amygdala in young male adult rats (Bolton et al., 2018) or following chronic treatment with antidepressants, such as vortioxetine and fluoxetine, starting in young adulthood in female mice (Liu et al., 2021; Zheng et al., 2024). While these studies demonstrated that ELA-induced behavioral deficits can be prevented or reversed, their suitability as a potential clinical intervention varies. Manipulation of the CRH system centrally to demonstrate a specific mediator of ELA is a sound research strategy but ultimately is not scalable as a potential translational intervention. Improved maternal diet can be translated for application clinically. Given the variation in the timing of, or partaking in, breastfeeding, however, it is unclear the extent to whether such an intervention would impact their children. Treatment with antidepressants or TrkB agonists are more clinically relevant, but only target certain circuits in the brain. In the present study, we focused on mitochondria, key subcellular targets of chronic stress in mature and developing systems (Picard et al., 2014; Hoffmann and Spengler, 2018; Picard and McEwen, 2018; Daniels et al., 2020). This focus on mitochondria also provides future opportunities to determine whether systemic administration of antioxidants can impact peripheral systems that have been reported to exhibit dysfunction due to ELA (Birnie and Baram, 2025). Initial responses to ELA include mitochondrial hypermetabolism, reflected in increased mitochondrial oxidative phosphorylation (OXPHOS) capacity and increased activity of individual complexes in the electron transport chain (Nold et al., 2019; Eagleson et al., 2020; Ruigrok et al., 2021b; Gyllenhammer et al., 2022). This likely represents an initial positive adaptation to increasing energy demands associated with stress (Picard et al., 2018). Such increases, however, particularly those associated with complex I, lead to an overproduction of reactive oxygen species (ROS; Liu et al., 2002; Murphy, 2009). In the absence of an adequate antioxidant defense, increasing levels of oxidative stress over time reduce mitochondrial function (Yakes and Van Houten, 1997), as is observed in the hippocampus and hypothalamus of adult mice following ELA (Eagleson et al., 2020; Ruigrok et al., 2021b). Of interest, in male hippocampus, which exhibits a more limited impact of ELA on adult mitochondria than in females, proteomics revealed a sex-selective elevation of endogenous antioxidant protein species (Eagleson et al., 2020). Thus, the combination of factors aligned with our focus on antioxidants as an intervention target for normalizing oxidative stress.

MitoQ, a mitochondrial antioxidant, was selected as the intervention in the current study as it has a demonstrated safety profile in adult clinical trials (Gane et al., 2010; Snow et al., 2010; Williamson et al., 2020; Broome et al., 2021; Linder et al., 2024). Further, following long-term administration in wild-type mice, MitoQ had no impact on mitochondrial function, mtDNA copy number, whole-body metabolism, food and water intake, or performance on cognitive and motor function (Rodriguez-Cuenca et al., 2010; McManus et al., 2011; Stucki et al., 2016). MitoQ has been shown to improve brain oxidative stress status, mitochondrial function, or behavior in rodent preclinical disease models (McManus et al., 2011; Davies et al., 2013; Feillet-Coudray et al., 2014; Fink et al., 2014; Ahmed et al., 2015; Stucki et al., 2016; Bai et al., 2023). Importantly, MitoQ can be administered in drinking water, avoiding the stress associated with intracerebral, intraperitoneal, and intravenous injections or oral gavage. Further, clinical trials using MitoQ reported improved vascular function in healthy older adults and those with peripheral artery disease, reduced serum liver enzymes in patients with chronic hepatitis C, and attenuation in exercise-induced increases in serum F_2_-IsoPs levels and muscle mitochondrial DNA damage but had no impact on Parkinson's disease progression (Gane et al., 2010; Snow et al., 2010; Rossman et al., 2018; Park et al., 2020; Williamson et al., 2020; Broome et al., 2021). Positive and negative effects for dTPP alone have been reported in animal and in vitro models, including cell disruptions at higher doses, with effects attributed to the dTPP cation rather than antioxidant activity (Leo et al., 2008; Reily et al., 2013; Huang et al., 2015; Trnka et al., 2015; Bond et al., 2019; Le Gal et al., 2021). We therefore chose a dose of MitoQ (100 μM) previously shown to exhibit improved cognitive function in a mouse model of Alzheimer's disease at which dTPP had no effect (McManus et al., 2011). We demonstrated that treatment with MitoQ but not dTPP at this dose improved adult hippocampal complex I activity. In contrast, both agents improved performance in the sucrose preference task, although there was significantly more heterogeneity in the dTPP response. Mechanisms of action for the impact of dTPP in some reports have suggested changes in mitochondrial membrane potentials that occur with enhanced permeability may affect certain phenotypes (Reily et al., 2013; Trnka et al., 2015; Gottwald et al., 2018; Bond et al., 2019). The very large heterogeneity of sucrose preference outcomes in the ELA + dTPP group, compared with the ELA + MitoQ group, indicates an inconsistent impact of the carrier on the cells in circuits mediating this specific rewarding behavior. It is important to emphasize that the MitoQ treatment groups for both complex I and anhedonia normalization exhibited limited variance and thus, the clear choice for mitigating oxidative stress. Future dose–response studies are warranted to determine whether a lower dosing of MitoQ/dTPP can effectively normalize specific behaviors due to ELA while eliminating the dTPP effect.

Many preclinical studies initiated MitoQ treatment either at the same time or prior to an insult that induces a pathological state, with the goal of mitigating increases in oxidative stress and subsequent negative sequelae observed in the absence of MitoQ (McManus et al., 2011; Xing et al., 2019; Haidar et al., 2022; Huang et al., 2022; Olajide et al., 2022; Gomez-Deza et al., 2023; Hu et al., 2023; Bannon et al., 2024; Böhm et al., 2025; Zhao et al., 2025). These studies typically focus on understanding the role of oxidative stress in disease etiology or the identification of preventative interventions in clinical populations. We note that while MitoQ treatment in the current study is initiated at weaning, well after the ELA period, the phenotypic measures used in the present study emerge after weaning. Specifically, decreases in sucrose preference following LBN are observed in adult, but not adolescent female mice (Goodwill et al., 2019; Ma et al., 2025). Similarly, reduced mitochondrial complex I activity occurs in adult, but not juvenile, hippocampus (Eagleson et al., 2020). Thus, while a longitudinal treatment study would be needed to determine reversal versus prevention, we suggest that the treatment paradigm likely represents the latter rather than rescue of these adult outcomes. Improvements in behavioral dysfunction also have been reported when MitoQ treatment is initiated after the onset of overt symptoms in mouse models of Alzheimer's disease, aging, and spinocerebellar ataxia type I (Stucki et al., 2016; Young and Franklin, 2019; Olesen et al., 2020). Future studies can determine whether starting this intervention in adults can reverse mitochondrial and behavioral dysfunction observed following exposure to ELA.

The present study demonstrates an association between mitigating oxidative stress and improved hippocampal mitochondrial function and sucrose preference. A role for the ventral hippocampus in sucrose preference has been reported, with an emphasis on projections to the nucleus accumbens (Bagot et al., 2015; Sambolín-Escobales et al., 2022; Kwon et al., 2025). For example, female-specific deficits in sucrose preference following adult subchronic variable stress were prevented by DREADD-mediated inhibition of the ventral hippocampus to nucleus accumbens pathway; conversely, reduced sucrose preference was induced in males when this pathway was activated during the stress protocol (Williams et al., 2020). Based on these adult studies, it is tempting to suggest that there is a direct link between the impact of MitoQ on hippocampal mitochondrial function and increased sucrose preference in females exposed to ELA. What might the improvement in complex I activity represent in males? Cognitive deficits are also observed following ELA, although many studies focused only on males (Brunson et al., 2005; Rice et al., 2008; Wang et al., 2011; Molet et al., 2016; Ruigrok et al., 2021b). When both sexes were included, studies indicate that males may be more impacted in tasks requiring an intact dorsal hippocampus (Naninck et al., 2015; Bath et al., 2017). Future treatment studies are necessary to determine whether the improved mitochondrial function observed in male hippocampus following MitoQ will be paralleled by improved cognitive performance.

## Synthesis

Reviewing Editor: Sam Golden, The University of Washington Decisions are customarily a result of the Reviewing Editor and the peer reviewers coming together and discussing their recommendations until a consensus is reached. When revisions are invited, a fact-based synthesis statement explaining their decision and outlining what is needed to prepare a revision will be listed below. The following reviewer(s) agreed to reveal their identity: T Chase Francis.

Dear Dr. Eagleson,

It has been very difficult to identify a second reviewer for your submission, I appreciate your patience. I have decided to move forward with comments based on one expert reviewer, who found your manuscript of interest and has suggested appropriate revisions. I will take an active role in reviewing the rebuttal in lieu of waiting to identify a second reviewer and further delaying your ability to respond to the comments. Below you will find the comments, should you like to revise your manuscript for re-review, please be sure to address them on a point by point basis.

Reviewer 1:

The authors present the effects of an early life adversity (ELA) model on hippocampal mitochondrial complex I function and behavior. The authors treat ELA mice with treatments aimed to enhance mitochondrial function during this ELA. There are a few statistical comparisons lacking in the manuscript. The primary concern is the differential effect on male and female mice at baseline within the ELA which makes direct comparisons difficult. See comments below:

• Post-hoc comparisons between treated and non-treated groups should be conducted, not just between sex comparisons, especially between CAU vehicle and ELA vehicle or ELA treatment. This outcome could be due to the ineffectiveness of the ELA paradigm on male animals in sucrose preference and so males are not the appropriate comparison. In addition, there needs to be an additional group to compare CAU vehicle with CAU treatments (dTPP and/or mitoQ) in bodyweight, sucrose preference, and complex I activity.

• The ELA model appears to affect hedonic outcomes in females only both in the data presented and the literature cited. The introduction should frame this as a sex-specific ELA/stress paradigm. Please also provide a comment in the discussion on the predicted reason as to why ELA did not affect sucrose preference in male mice.

• While raw weight gain is indeed informative, changes in weight gain (%) for each animal over time would be beneficial for a within-groups measure.

• Error bars are needed on each of the figures.

• Sucrose consumption behavior is typically used to measure loss of pleasure or to measure the rewarding effect. In Figure 4, it difficult to tie this to anhedonia because there is a complex I deficiency in ELA females as shown in Figure 3. Therefore, it is not possible to discern that decreased sucrose consumption is a sign of a depression-like phenotype or simply a mean decrease in intake of calories due to decreased metabolism.

Minor

• Individual points need to be labeled in the figure, not just in the legend. Label red as female and blue as male.

• Divide the sentence at "because" in line 81-87 to increase readibility.

• Consider removing "which is a key generator of free 91 radicals that produce oxidative stress" in line 90-91 due to repetition in line 71-72.

• Line 263: Add "of" between "the impact" and "ELA".
