# Supplementary figures and images for: Treatment of Mitochondrial Disturbances due to Early Life Adversity in Mice Results in Restoration of Complex I Activity and Normal Reward Behavior
Source: eNeuro. 2025 Sep 24;12(9):ENEURO.0172-25.2025. doi: 10.1523/ENEURO.0172-25.2025 (PMC12469007; doi:10.1523/ENEURO.0172-25.2025)

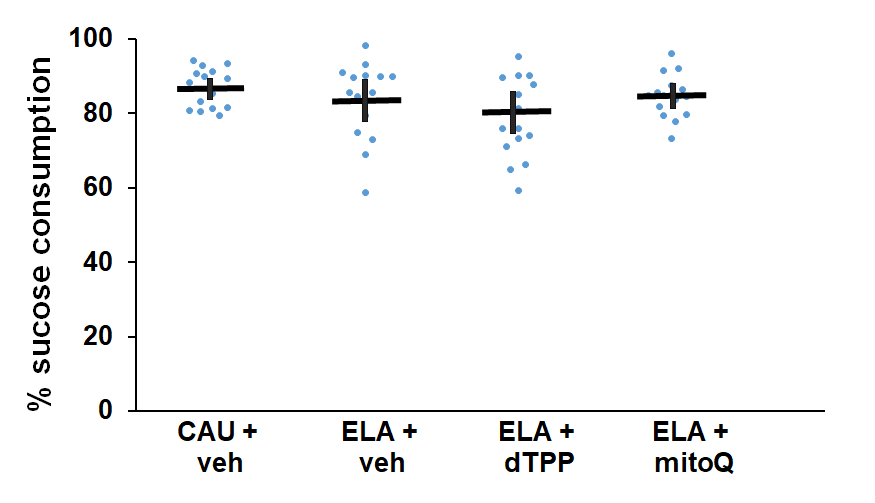

Supplement: Figure 5-1 — Early life adversity (ELA) treatment has no impact on sucrose preference in male mice treated with vehicle (veh), dTPP and MitoQ compared to care-as-usual (CAU; Kruskal Wallis test, H(3) = 4.763, p = 0.190). The raw data contributing to this figure are included in Figure 5-2. Each symbol represents an independent sample, with the bars representing the group mean ± 95% confidence interval. Download Figure 5-1, TIF file. [file eneuro-12-ENEURO.0172-25.2025-s005.tif]
